# The Rising Burden of Diabetes-Related Blindness: A Case for Integration of Primary Eye Care into Primary Health Care in Eswatini

**DOI:** 10.3390/healthcare9070835

**Published:** 2021-07-01

**Authors:** Sharon Nobuntu Maseko, Diane van Staden, Euphemia Mbali Mhlongo

**Affiliations:** 1 Department of Optometry, School of Health Sciences, University of Kwa-Zulu Natal, Durban 4001, South Africa; wallaced@ukzn.ac.za; 2 Department of Nursing, School of Nursing and Public Health, University of Kwa-Zulu Natal, Durban 4001, South Africa; mhlongoem@ukzn.ac.za

**Keywords:** primary health care, primary eye care, health services integration, diabetic retinopathy, vision impairment

## Abstract

There is a rampant increase in diabetes prevalence globally. Sub-Saharan Africa (SSA) is projected to carry the largest burden of diabetes (34.2 million) by 2030. This will inevitably cause a parallel increase in diabetes-associated complications; with the predominant complications being blindness due to diabetic retinopathy and diabetic cataracts. Eye programs in developing countries remain inadequate, existing as stand-alone programs, focused on the provision of acute symptomatic care at secondary and tertiary health levels. Over 60% of people with undiagnosed diabetes report to eye care facilities with already advanced retinopathy. While vision loss due to cataracts is reversible, loss of vision from diabetic retinopathy is irreversible. Developing countries have in the last two decades been significantly impacted by infectious pandemics; with SSA countries committing over 80% of their health budgets towards infectious diseases. Consequently, non-communicable diseases and eye health have been neglected. This paper aimed to highlight the importance of strengthening primary health care services to prevent diabetes-related blindness. In SSA, where economies are strained by infectious disease, the projected rise in diabetes prevalence calls for an urgent need to reorganize health systems to focus on life-long preventative and integrated measures. However, research is critical in determining how best to integrate these without further weakening health systems.

## 1. Introduction

Diabetes prevalence is on the rise globally [1,2]. This is of concern as diabetes is a serious chronic metabolic disease which, in clinical practice, has the greatest impact on health compared to any other non-communicable disease (NCD) [3,4]. Long-term effects of this condition lead to macro and microvascular damage [5]. Consequently, a predominant complication of diabetes in the eye comes in the form of diabetic retinopathy (DR). Another common complication is diabetic cataracts. Both of these conditions lead to loss of vision, including blindness [3]. While vision loss due to cataracts is mostly correctable, loss of vision from DR is irreversible. Of concern is that as the prevalence of diabetes increases globally, there will be a parallel increase in complications associated with this condition, including the aforementioned potentially blinding complications. Approximately 90% of the world’s blind people live in developing countries [6,7]. Cataracts and refractive errors together account for up to 75% of unnecessary blindness in low-and-middle income countries (LMICs). These two conditions are also the most straightforward and cost effective to manage; minimal screening is required, treatment outcomes are often immediate and satisfactory (with surgery or spectacles, respectively), and little follow-up is needed [8]. Eye care programs in developing countries have therefore placed much attention on addressing these two common causes of vision impairment [9]. However, eye services in these countries remain inadequate since eye diseases such as cataracts and refractive errors are often perceived as non-life-threatening and therefore receive little priority from key decision makers [10].

Eye care programs in LMICs have historically been established as separate stand-alone entities, poorly integrated into the general health system [11]. A contributing factor to this may be because cataracts and refractive errors do not require much input outside of eye health; an anaesthetist, for example, is not required during adult cataract surgery [11]. Eye care service delivery has also been predominantly disease-specific and focused on acute symptomatic care rather than offering life-long, preventative and comprehensive care [12].

There is now growing evidence of a global epidemiological transition from infectious to chronic non-communicable diseases (NCDs) as causes of mortality [13,14]. Diabetes is one of the four most prevalent NCDs globally, in addition to cancer, cardiovascular and respiratory diseases [15,16]. Four out of every five adults with diabetes live in developing countries [17]. According to International Diabetes Federation estimates, there will be a 98% increase in the number of people diagnosed with diabetes in Africa between 2010 and 2030, with the SSA region projected to carry the largest burden (34.2 million) [18,19]. This region has also been significantly impacted by infectious diseases in the last two decades, committing more than 80% of their health budgets towards infectious conditions within this period [19]. Consequently, NCDs have remained neglected despite the fact that they account for over a quarter of the disease burden in these settings. NCDs are now therefore emerging as a silent killer [20,21]. 

The projected increase in the burden of diabetes, especially in SSA, brings new and difficult challenges which most eye care programs are not equipped to tackle [9]. Treatment of DR is expensive for both the patient and service provider, often with poor outcomes. Unlike cataracts and refractive errors, the management of a diabetic eye patient requires a comprehensive life-course and multidisciplinary approach. Up to 80% of people living with diabetes are in LMICs, with an estimated 93 million people worldwide living with DR [22]. This implies that the bulk of the 93 million people with DR can also be expected to be in developing countries. A major risk factor for DR is the duration of diabetes. Diabetes-related eye complications occur progressively and are symptomless until advanced stages. Every diabetic will likely have some degree of retinopathy after 10 or more years of living with the condition. However, undiagnosed or poorly controlled diabetes, as well as comorbidities such as high blood pressure and high cholesterol will speed up progression of DR [22]. Therefore, in order to prevent loss of vision, diabetes must be diagnosed and controlled early, and diabetic eye complications must be detected even before diabetic patients themselves realize that there is a problem [9]. It is for these reasons that primary prevention has been highlighted as the most effective way of controlling this epidemic and its adverse socio-economic effects [23]. 

Primary health care (PHC) is the first point of contact in a health system for most patients. It focuses on providing community-based health services that are equitable, comprehensive, affordable and accessible to all, throughout the life-course of an individual [24]. PHC places emphasis on health promotion and disease prevention. Primary eye care (PEC) is an integrated component of PHC [25]. Evidence shows that PHC and PEC are inadequate in developing countries; often focusing on the provision of acute symptomatic care [26]. As a result, many patients will have already experienced a loss of vision by the time they are screened for DR. As much as one-third of individuals already have DR at the initial diagnosis of diabetes in these settings and in Africa, up to 25% of type 2 diabetics already have DR at the time of the initial diagnosis of the diabetes [27]. 

Another common eye complication of diabetes is early onset cataracts [4,18]. Cataracts occur due to the clouding of the natural lens of the eye usually, as a result of the normal ageing process. As people live longer, the prevalence of this condition will increase. Diabetes, however, causes a predisposition to premature cataracts. Although there is limited understanding of the exact pathophysiology of diabetic cataracts, what is certain is that the condition causes a five-fold increase in the occurrence of early onset cataracts [28]. Cataract blindness is mostly reversible through a simple, low-cost surgical procedure; and surgical outcomes are often immediate and satisfactory for both the patient and service provider. However, due to weak eye health systems, particularly PEC services, the burden of this condition remains high in developing countries and may be exacerbated by the rising public health burden of diabetes [10,29].

Catastrophic health conditions which have challenged most developing countries over the years have resulted in an increase in externally funded and led interventions aimed to combat them. This has in turn led to the disempowerment of governments and a rise in interventions targeting specific health conditions within health systems [30]. Although the quality of a health system is partly dependent on some of its components being specialized, in many LMICs where health systems are weak and where interventions compete for limited resources, such specialization in its extreme form has caused dire fragmentation of health services (including PHC services) with resources, activities and staff contained within a series of silos [30]. Indeed, some vertical programs such as HIV, TB and malaria have benefited from this through garnering much attention and support from international donors and decision makers. However, this has led to the neglect of other seemingly less-pressing conditions such as NCDs and eye health. The projected escalation in the burden of chronic NCDs such as diabetes in developing countries, as well as their associated complications is therefore concerning, as health systems in these countries are unprepared to deal with these conditions. 

Resources for the treatment of diabetic eye complications are expensive and the prognosis is poor, therefore the prevention of this condition through decentralization and the integration of eye services into existing primary care services is key. The purpose of this narrative review is therefore to highlight the growing challenge of diabetes-related blindness on eye care programs in developing countries, and the potential benefits of integrating primary eye care into primary health services.

## 2. Methods

A literature search was conducted between 2019 and 2020 using the following electronic databases: MEDLINE, PubMed, EMBASE and Google Scholar. The search was based on combinations of keywords that included primary health care, primary eye care, eye care services, eye health, developing countries, low- and medium-income countries, integrated health services, health service integration, non-communicable diseases, diabetes, blindness, visual impairment. In addition to this, citations from the reference lists of articles guided the search as well as desktop reviews of official documents and reports released by the Eswatini Ministry of Health. Only papers written in the English language were included. A total of 71 papers were reviewed and included in this evidence synthesis paper.

## 3. Results

### 3.1. The State of Eye Health in Developing Countries

Health systems in SSA are strained by life-threatening infectious diseases. Eye health has therefore been a neglected component of health care in developing countries. Most developing nations have only recently started to establish dedicated national eye health policies. In line with WHO recommendations, however, member states have, over the years, embarked on establishing and strengthening blindness prevention programs, with a particular emphasis on cataracts and refractive errors. This is because these are two major causes of unnecessary vision impairment which are relatively simple and cost effective to treat [9]. Despite this, the burden of these conditions remains high in developing countries where eye care programs are often overshadowed by other pressing initiatives. Consequently, eye care services, and especially those for the detection and management of more severe ocular problems such as DR, remain rudimentary and confined to urban areas [31].

Studies conducted in Kenya, Eswatini and Nigeria, found a delayed presentation of both diabetes and DR as a major challenge. Approximately 50% of people in these settings live with undiagnosed diabetes and over 60% report to eye health facilities with already advanced DR and poor prognosis [4,32,33]. Evidence from high-resource settings shows that a well-established program for routine DR screening is effective in preventing vision loss. England, for example, has attained over 80% DR screening coverage; this is mainly attributed to DR screening appointment reminders which are periodically sent out to diabetic patients [34]. In LMICs, however, where the bulk of the population lives in rural outskirts and where barriers to eye services such as distance, cost, and lack of awareness/priority are rife, such an initiative may not be as successful. Therefore, in disadvantaged settings, the importance of decentralization and the integration of eye services into PHC is key to preventing diabetes-related vision impairment. 

Over 75% of blindness in developing countries is preventable and cataracts account for 50% of cases [35]. Findings from a study in Nigeria revealed that almost 50% of participants had undergone a traditional procedure for cataract removal (couching), with 75% of these eyes subsequently going blind [36]. This highlights the existing gaps in cataract (and general eye care) service delivery in LMICs. The natural lens of the eye is transparent and situated on the internal anterior aspect of the eye. Any clouding of this lens (as occurs with cataracts) obscures all other structures located posterior to it—which makes the monitoring and detection of a pathology occurring at the back of the eye difficult or impossible. If not prevented or detected early, the majority of eye disorders that occur posteriorly in the eye, such as DR, often lead to irreversible loss of vision. While diabetes may further increase the already high burden of cataracts in developing countries, cataracts may in turn exacerbate the burden of DR by making the early detection of this condition difficult. If not addressed, the increased burden of diabetes-related blindness may counteract all efforts aimed at addressing unnecessary blindness as a public health problem. This in turn, could undermine other broader global strategies such as the Sustainable Development Goals; especially those focused on poverty and hunger reduction, equality, health, education, as well as work and economic growth [37]. A further challenge is that the ageing population in SSA is growing, hence there is a projected increase in patients who will be affected by cataract and diabetes.

**Figure 1:**
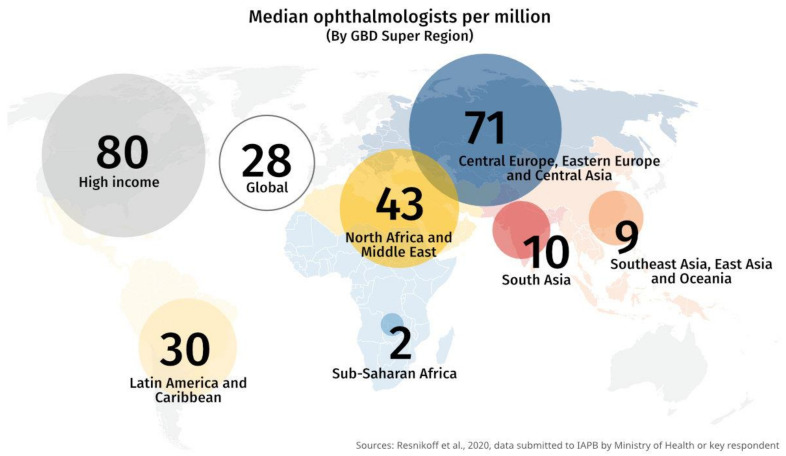
Highlighting the shortage of ophthalmologists in SSA compared to other regions [40].

A study conducted in Tanzania revealed that eye health policies were of little priority to the government of this country, with limited resources allocated to eye health. A key barrier to the establishment of an effective eye health system in Tanzania was said to be a “lack of perceived urgency towards eye health due to the non-lethal nature of eye diseases [38].” Consequently, eye care across this country is largely provided by non-governmental organizations (NGOs), which includes eye health financing. This situation is similar across a majority of eye care programs in developing countries where there is a lack of government ownership and sustainability of eye services. A review conducted by Palmer et al. in 2014 found that out of 21 SSA countries, only 5 met WHO standards for eye surgery personnel with similar projections made for the year 2020 [39]. The inadequacy of human resources for eye health (HReH) remains significantly high in SSA in comparison to any other region. Figure 1 below depicts this, with ophthalmologists (per million population) as an example.

Most if not all systemic chronic conditions inadvertently affect the eyes. As a result, there is a widening gap between the need and supply of eye health services [18]. Malawi for example, having a population of approximately 15.2 million, has seven practicing ophthalmologists, no retinal imaging equipment and two laser machines. Most eye patients are reportedly seen by ophthalmic clinical officers who have limited training on retinal examination and diagnosis [18]. Eswatini, with a population of 1,093,238, has three ophthalmologists, five public sector optometrists and 12 eye nurses; all of whom are located in urban areas. The country has one retinal imaging machine and two laser machines also confined to urban settings and no HbA1c testing mechanism in the public sector [41]. HbA1c is a measure of how well controlled ones’ blood sugar has been over a period of about 3 months [35]. Furthermore, the eye care program in Eswatini largely exists as a stand-alone entity, poorly integrated into the general health system. This country’s national eye care plan has not yet been integrated into the national health strategic plan [41]. 

The unavailability of ophthalmic equipment is often another major limitation to eye service delivery, especially in the public sector of LMICs [42]. An ophthalmic equipment survey conducted in 173 health care settings in Africa and Asia found that over 60% of services did not have a photocoagulation laser machine—key equipment for treating DR. Another survey recently conducted in Nigeria revealed that only 30% of ophthalmologists had access to laser machines in that country [43,44]. 

### 3.2. A Focus on Eye Care Services in The Kingdom of Eswatini

The Kingdom of Eswatini is a landlocked country in Southern Africa with an estimated land area of 17, 364 square kilometres. It has a homogenous population of 1,093,238, 63% of which lives below poverty line, and 29% living below the extreme poverty line [45]. The poverty level has been associated with a high burden of both non-communicable and communicable diseases. Based on a gross domestic product (GDP) per capita of approximately USD 3000, Eswatini is classified as a lower middle-income country [45]. As a country reported to have the highest HIV/AIDS prevalence in the world (27.2%), Eswatini, with assistance from various global partners, has placed immense focus on trying to curb this pandemic and its socioeconomic challenges. Since the year 2000, a number of large Global Health Initiatives have resulted in a concerted response towards combating this [46]. However, such GHIs have often brought in disease-specific funds along with intensely focused mechanisms, tight conditionalities and strict deadlines. This has overwhelmed and distracted key decision makers from other responsibilities such as NCDs. As such, the country has an escalating number of individuals living with often poorly managed NCDs amidst a health system that is battling with infectious diseases and unprepared to address NCDs and their associated complications [47]. 

One in four people die in Eswatini due to NCDs [26,48]. According to Eswatini’s Services Availability Mapping Report (SAM), “The country is still struggling with respect to NCDs, yet they are among the leading causes of morbidity and mortality.” This report further recognizes diabetes as part of the top 10 leading causes of ill health in the country and highlights investment in its prevention as critical [49]. Results from the country’s latest STEP survey (2014) show an estimated diabetes prevalence of 14.2% in Eswatini [50]. The latest Essential Health Care Package report states that diabetes is currently the fifth leading cause of mortality and the eighth leading cause of morbidity in the country. This condition is also the third leading cause of NCD visits to the country’s outpatient departments following hypertension and musculoskeletal conditions. It is for this reason that diabetes falls under the Government of Eswatini’s list of priority diseases [49]. 

Eswatini has prioritized health services according to disease burden as reflected by leading causes of hospital visits from the Health Management Information System (HMIS). The current priority diseases (in order of priority) are HIV/AIDS and other sexually transmitted diseases (STDs), TB, cardiovascular disease (CVD)/hypertension, diabetes, oncology, family health, and basic curative services and emergencies [49]. What is of note is that despite the rising prevalence of diabetes and NCDs in the country as highlighted in the 2017 SAM report, eye health does not form part of this list, even though the majority of the priority conditions result in eye complications. Eswatini’s 2017 NCD Program report projects eye disorders as one of the leading causes of the country’s economic pressures in the near future, however, eye health does not appear in the list of conditions that the NCD Program’s Strategic Plan focuses on [50]. The exclusion of eye health has significant consequences because if not managed effectively, the burden of irreversible visual impairment including blindness could rise. This is especially true for diabetes-related complications. Although some achievements have been made, eye services in Eswatini remain limited and continue to be overshadowed by other health initiatives. Eye care services receive no direct budget allocation from the Ministry of Health. The service is funded mainly through income generated from spectacle sales as well as occasional donor funding. Human resource for eye health also remains a challenge. Approximately 30 eye nurses were trained over the years; however, only seven remain active in the country with the rest having been lost to career progression outside of eye health. The WHO recommended minimum ratio of ophthalmologist: population is 1:250,000, Eswatini has a ratio of 1:400,000. With respect to public sector optometry, there is one optometrist per 240,000 people, which is well below the WHO recommendation of one optometrist per 50,000 persons. Eye care services are only available at the third and fourth tier of service delivery, i.e., at the regional and national referral hospitals, all located in urban areas [49]. Therefore, the service is currently being accessed by only 20% of the Swazi population [45]. The consequences of this are anecdotally evidenced in the NCD program report findings, as well as in the increasing number of patients reporting to the country’s eye facilities with advanced diabetic complications. In a retrospective review of patients with diabetes seen at one of the mission eye clinics in Eswatini, 76% had advanced proliferative diabetic retinopathy and 38% were blind (visual acuity <3/60) at initial presentation [33]. 

### 3.3. The Role of Primary Health Care

The 1960s–1970s saw a global drive towards population health as a solution to social and economic growth in LMICs. This culminated in the adoption of the Alma-Ata declaration in 1978 which was met with much support and enthusiasm. This declaration focused on “health for all by the year 2000” and propagated PHC strengthening as a solution to attaining population health. Although relevant, the goal of the Alma-Ata declaration was never achieved [51]. Two decades later, its ideals were re-introduced as the Millennium Development Goals (MDGs) whose attainment also relied on PHC strengthening. The MDGs were therefore accompanied by the release of the 2008 WHO report entitled “Primary health care: now more than ever.” By the year 2015, however, the objectives of the MDGs had still not been met by many developing countries; with the WHO attributing this to the lack of integrated health services in LMICs [52]. Consequently the “WHO Global strategy on people-centred and integrated health services” was developed and adopted in 2015. This strategy called for a major shift in the way health services were being funded, managed and delivered [53]. Despite all the above global efforts pointing towards its importance, PHC remains inadequate in developing countries. With the emerging burden of chronic NCDs amidst health systems that are already overstretched and strained by infectious conditions, the values of PHC and integrated services therefore need to be re-visited.

Results from a recent national blindness and visual impairment survey conducted in Nigeria revealed that nearly half of all participants with high blood sugar levels did not know they had diabetes [4]. Reports from studies conducted in Kenya suggest that as many as 50% of people with diabetes in this country may be undiagnosed [32]. It is during this period of untreated diabetes that the constantly high blood sugar causes microvascular damage leading to complications including DR. As is the case with a majority of NCDs, high blood sugar levels in diabetes develop gradually over many years of cumulative exposure to risk factors. They are initially not severe enough for a person to experience any symptoms hence individuals in developing countries often live for many years with undiagnosed diabetes [5]. The progressively high blood sugar levels eventually lead to disease manifestation from the third decade of life [24,54]. There is therefore up to three decades during which PHC can play a role in the prevention of disease within communities. Once one has been diagnosed with diabetes, there is usually a further ten to twenty-year lag before the individual develops diabetic complications such as DR and cataracts, a period which provides another opportunity for secondary disease prevention at the PHC level [4]. Therefore, whether preventative action is taken or not during these latent periods will impact public health systems in future [15]. 

### 3.4. The Role of Primary Eye Care

Like PHC, PEC services provide a first point of contact into an eye health system. PEC therefore provides a platform for prevention, early detection and the timeous management of eye disorders [4]. Minor eye conditions such as allergic and infectious conjunctivitis, dry eye and simple forms of refractive errors can also be easily treated at the primary level. None of the PHC nurses in Eswatini are trained in PEC [41]. In LMIC, PEC remains inadequate or non-existent as the focus is often on providing curative eye services at the secondary and tertiary levels of care [10]. This leads to the late presentation of cases which could have otherwise been prevented or detected and managed early. Moreover, the lack of PEC results in higher levels of care becoming burdened with minor eye conditions, leaving little room for adequate management of complicated cases such as DR and cataracts. In a study conducted in Nigeria, patients with red or itchy eyes were found to most likely visit a hospital for treatment, whereas those with painful eyes were most likely to visit the nearest pharmacist [55]. Such health seeking behaviour results in eye specialists spending valuable clinic time treating the aforementioned eye conditions which are often treatable at the primary level at the expense of more serious cases [38]. 

Mobile eye care clinics have been instrumental in reaching rural communities in developing countries. This has been done through eye care teams which visit communities every 2 to 24 months, providing eye care, including surgery where possible. Although having significant impact, a number of challenges with mobile eye care clinics have been cited. The major ones being a lack of government ownership since these clinics are often funded and conducted by non-governmental organizations. Consequently, issues of sustainability, patient follow-up care and the failure of mobile teams to handle massive patient turnouts arise [56]. 

The WHO released the 2014–2019 “Global Action Plan for Universal Eye Health.” This document aimed to create a road map towards decreasing the burden of avoidable blindness including its associated limitations on quality of life and economic growth in developing countries [57]. There are five principles which underpinned the 2014–2019 Global Action Plan for universal eye health, namely: universal access and equity; human rights; a life-course approach; the empowerment of people with vision impairment; and evidence-based practice. PEC forms the basis of four out of the five principles. The recently published World Report on Vision further promotes “integrated people-centred eye care” (IPEC) as a core approach to address the spectrum of challenges faced by countries in the delivery of eye services and the projected strain that this challenge will have on general health systems. IPEC is defined as eye care services that are managed and delivered to assure a continuum of promotive, preventive, treatment and rehabilitative interventions against the range of eye conditions, which are coordinated across the different levels and sites of care within and beyond the health sector, according to needs throughout the life course. The health-system approach adopted by IPEC encompasses four strategies, two of which focus on primary care. These are “engaging and empowering people and communities” as well as “reorienting the model of care based on strong primary care [7].” 

### 3.5. Primary Eye Care in Sub-Saharan Africa

In SSA, PEC is defined as the provision of basic eye services that are integrated within the PHC system. These services are therefore offered by full-time PHC workers based at the lowest level of health care and include the diagnosis and treatment of minor eye conditions as well as the referral of more complex cases [58].

The importance of PEC strengthening as a strategy to improving access to eye care services is well documented [59,60]. However, limited evidence is available from across Africa. Major challenges noted include the following:

- Lack of a unanimous definition of PEC and a lack of clear guidelines on technical eye-related skills required by PHC workers. Consequently, there is disagreement on the type of equipment and consumables needed as well as the scope of training, support and supervision necessary for PEC [61].
- Lack of appropriate PEC skills and low productivity amongst PHC workers, diminished trust in the PEC services by targeted communities.
- Common causes of vision impairment, i.e., (immature) cataract, glaucoma, DR and refractive errors are often beyond the capacity of the general doctor or nurse. Equipment required by an eye specialist to diagnose these conditions is not available at PHC level [58]. The use of mobile eye care teams may assist in bridging this gap. A study in South Africa, for example, found that a technician who visited PHC facilities with a mobile camera was not only able to detect DR but also cataract cases needing surgery [62].
- Despite improved supervision, “persistent deficiencies in the diagnosis and treatment of common eye conditions” was found to be a major problem across three SSA countries (Kenya, Malawi and Tanzania) [58]. Courtright et al. stated that it is important for supervisors to have technical skills that match those required of the PHC worker. External support is also highlighted as beneficial in the provision of basic equipment and technical training [61].

To address some of the above challenges, in 2018, the WHO Africa office developed a package of PEC interventions for the Africa region. As a follow-up to this, a “Delphi Survey” was conducted to determine the technical capacities required to deliver the package in health facilities in SSA. Results from the survey revealed that “insights into the technical complexities of PEC and the technical capacity required to deliver this service were lacking in SSA.” The scaling up of human resource for health as well as strong government support and partnerships are mentioned as crucial in the implementation and sustainability of PEC services in the region. However, the importance of conducting a feasibility study before implementing a new health intervention is emphasized [63].

### 3.6. Integration of Primary Health Care and Primary Eye Care Services

Integrated care describes the extent to which patient-centred strategies are coordinated across the various functions and structures of a health system [64]. In order to address the burden of diabetes-related visual impairment, there is an urgent need to move towards decentralization of basic eye services as well as integration of these into general PHC services. One of the key objectives of the WHO’s Global Action Plan on avoidable blindness is “to encourage the development and implementation of integrated national eye health policies, plans and programs to enhance universal eye health, with activities in line with WHO’s framework for action for strengthening health systems to improve health outcomes [8].” Critical shortages and the unequal distribution of human resources for eye health have been cited as among the main contributors to the problems of eye care service delivery, particularly in SSA [39]. Bringing eye care services closer to communities through the integration of eye health into existing PHC services has been put forward as a solution to improve the accessibility of this service. 

However, over the past decades, there has been much debate on the benefits of integrating programs aimed at controlling specific diseases (“vertical”), such as blindness prevention programs into general (“horizontal”) health care services [65,66]. This debate has largely been fuelled by the rigid dichotomy created around the terms “vertical” and “integrated/horizontal.” Although widely and loosely used, the meaning of these terms in practice is not as rigid and clear-cut. Moreover, the extent of verticality or integration will vary under different contexts and intervention—with differing advantages and disadvantages thereof [65]. 

Due to growing pressure on service delivery and health care financing in the United Kingdom (UK), the UK National Health Service (NHS) developed a plan that advocated for the greater integration of health services. Integration was highlighted as a solution towards creating an efficient and sustainable health and social care system, with increased attention on the prevention and public health [67,68]. To accelerate this process, models of integration from around the globe were studied and in line with this, a systematic review was carried out by Baxter et al. This review aimed to determine the effects of integrated care on health systems in countries outside of the UK and it focused on studies carried out in developed countries between 2006 and 2017. Results revealed that models of integrated care were in fact complex and contained multiple elements which could be broadly divided into four categories, as shown in Table 1 below [69]:

The review further revealed that models exercise integration at different and often multiple levels according to specific settings and circumstances; hence, evaluating and determining which model is best is often a challenging and futile exercise. Although this study screened 13,323 unique citations and reviewed 153 eligible studies, solid evidence on the positive effects of integration was inconclusive. Elements of integration which had a positive outcome for one setting did not necessarily bear the same result for another, suggesting that integrated care may be best suited to specific patient groups, such as those needing complex care and should not be thought of as a blanket solution [69]. 

India bears over 20% of the world’s blind and visually impaired population. Although approximately 90% of blindness in this country is preventable through cost-effective treatment, the accessibility and availability of eye services remains a major challenge for the neediest communities. India has, however, taken significant strides in addressing these challenges, and with approximately 5000 cataract surgeries conducted per million population per year, it has one of the highest cataract surgical rates (CSR) globally [70]. However, one study conducted in India suggests that initiatives whose activities, staff and resources are strictly confined to addressing a specific intervention may not be effective in dealing with complex conditions such as diabetes [71]. For example, only 20% of ophthalmologists in India were found to have access to the diabetic case records of their patients, which highlighted the challenges of poor coordination and incoherence that come with siloed programs [71].

The integrated model of PEC services being delivered within general community and primary health centres was cited as being the most effective for raising awareness and delivering PEC to rural and underserved urban areas in India [72]. It is considered cost-effective as eye services are provided within already existing infrastructure and human resources, which also ensures that eye care personnel work under the guidance of trained medical officers. Despite this, the set targets for rolling out this model have not been attained. Contributing factors mentioned are the shortage of personnel trained in PEC; deprived facilities; inadequate financial support; unskilled and undisciplined staff; poor equipment maintenance; and a lack of priority for PEC. Regular monitoring visits by an ophthalmologist, the creation of performance indicators as well as the establishment of a management information system for reporting services have been recommended as solutions to some of these challenges [72].

The PHC and PEC strengthening and integration strategy is well publicized as key to addressing the high burden of unnecessary visual impairment in developing countries. However, efforts to deliver upon this strategy have mainly been focused on the provision of training to PHC workers in PEC as well as the task-shifting of eye health services to PHC workers [25]. Understanding the general health system through considering each of its six interconnected building blocks as well as how eye care services are delivered within and alongside each of these building blocks is as crucial [38]. Eye health for example is often said to be integrated into the general health management information systems (HMIS) of many developing countries. However, closer investigation often reveals that these systems are not adapted to capture the full spectrum of eye disorders. In some countries, as little as three eye conditions are captured by HMIS [38]. 

The review conducted by Palmer et al. highlights the major challenges of specialist human resource for eye health in SSA [39]. A study conducted in Tanzania further revealed that although the integration of PEC into PHC bore positive outcomes in terms of improved detection and the prompt referral of potentially blinding conditions at the primary level, the “interconnectedness of health system building blocks impacted on delivery of eye services.” PHC workers that were trained in PEC became demotivated due to lack of specialist eye personnel to provide on-going supervision and support. The lack of financial resources, bureaucratic delays in the procurement of essential eye care supplies with resultant shortages were also raised as major challenges [38,39]. 

Creil et al. recommend that in order to decide whether or not to integrate a disease control program into existing general health services, the following three questions should be considered [66]:

1. Is it desirable? Will there be an added valuable output from integrating an intervention into the general health service package?
2. Is integration possible? Would general health workers have the ability to perform the task appropriately and with a level of standardization? This is particularly a key question in many developing countries, where the health workforce is limited and where community and primary health care workers, in particular, are often overburdened with a range of activities targeting various interventions. This leads to poor quality of services and poor health outcomes [30].
3. “Is it opportune to integrate?” In other words, does the general health service have the capacity in terms of human resource, equipment and supplies, etc. to accommodate an additional activity? In some cases, one may argue that perhaps integration may constitute a strong case for maximizing the current capacity of a general health service. The need to thoroughly interrogate and address these questions is imperative to ensure that integration is sustainable and does not lead to the further weakening of a health system.

Perhaps another consideration would be which component is best for integration considering the six building blocks of a health system. The integration of eye health at the point of service delivery, for example, often maximizes the utilization of the service, however, existing systems within governments in LMIC often impede the efficient delivery of eye care services as was evidenced in Tanzania and India [38,72]. Nonetheless, the integration of PEC and PHC services can play a significant role in addressing many of the health challenges faced by developing countries through community participation and empowerment, as well as increasing accessibility to health care. 

## 4. Discussion

Diabetes does not only pose serious socio-economic repercussions on the already weak economies and health systems of developing countries, but also complicates treatment and weakens the prognosis of other co-existing morbidities such as HIV, TB and emerging infectious diseases such as COVID-19. Its effects therefore undermine efforts of other important global health initiatives; this includes the eradication of unnecessary blindness as a public health problem in developing countries. This narrative literature review sought to highlight the challenges that the growing burden of diabetes may exert on eye care programs in developing countries. It further sought to highlight the role that primary health care, primary eye care and the integration of these services may possibly have in addressing these challenges. 

SSA is faced with various socio-economic and health challenges which have overshadowed eye health. Most eye programs in this region and other LMICs are poorly integrated into the general health system. Eye health policies therefore seem not to be well integrated into general health policies. Consequently, opportunities such as those for eye health financing, advocacy, on-going training and the progression of eye health personnel, are missed. Eye services have also been delivered within a disease-specific care model; often concentrating on the leading causes of blindness (cataracts and refractive errors) as well as the provision of acute symptomatic care [12]. The escalating number of patients reporting to higher levels of care with advanced and irreversible loss of vision due to diabetes highlights the inadequacy of primary care in developing countries. 

Diabetes occurs gradually over decades of cumulative exposure; these time lags present windows of opportunity for patient education, screening and the prevention of the disease at primary health care level. After the fourth decade of life, every individual will need near spectacle correction; a service that can be delivered at the PHC level. A spectacle prescription will require review and possible renewal every after approximately two years. Integrated and comprehensive PEC services would therefore afford a chance for the regular monitoring/screening and management of other NCDs and NCEDs common in this age group.

The need for the integration of eye services into general health systems is eminent. For a PEC to be effective, the broader health system must be considered. There must be careful and contextual consideration of how eye health is aligned and integrated into each of the six health systems building blocks, from primary to tertiary levels of care. This is particularly imperative in chronic care where, for example, well-established referral pathways and mechanisms to encourage follow-up care such as SMS messages must be in place. Effective and inter-linked health information systems will ensure the monitoring of both patients and services.

By virtue of their specialized nature, eye care services often fail to withstand the lengthy budgetary and procurement processes that exist within government systems [62]. This in part has contributed to government eye services often operating autonomously and with heavy reliance on donor funds. The integration of eye health into the general health financing structure is critical however, the importance of establishing income-generating mechanisms within eye care programs is as vital. This will assist in bypassing some of the negating aspects of government systems and ensure that the momentum of eye service delivery is maintained. It is also important for such mechanisms to be adopted within government policies to ensure transparency and accountability. Income generated through spectacle sales in Eswatini, for example, has been instrumental in sustaining some of the public eye services in this country; however, there are no official systems in place within the government to support and audit this [41]. 

Specialist HReH remains a significant challenge in SSA, as the study by Palmer depicts [39]. While mobile eye care clinics have helped bridge the gap between urban and rural eye services, these are often run and funded by non-governmental organizations and are therefore not sustainable. Hill mentions the lack of government ownership as a threat to the continuity of mobile eye services. He highlights the need for governments to integrate this service through for example, training existing PHC staff in PEC as well as creating permanent posts for the specialist cadres involved in these teams [56].

Considering the projected rampant increase in diabetes prevalence, immediate and feasible solutions to address the challenges of HReH need to be explored. In a country such as Eswatini, where the International Monetary Fund is calling for an urgent reduction in the government wage bill, the creation of more specialist posts may be an insurmountable exercise. Task-shifting could therefore be the most feasible solution. Training existing medical officers in cataract surgery, for example, may aid in reducing the backlog of cataract cases and thus allow for screening an early detection of blinding conditions like DR. Such training can be conducted at tertiary facilities with assistance from local ophthalmologists, external support, or both. A reduction in workload through PEC strengthening will create room for this. As a long-term solution, the review and improvement of eye health curricula in training institutions for nursing and medical students may be necessary.

The lack of human resources coupled with the growing burden of diabetes further calls for advancements in health technology and infrastructure. Telemedicine in LMICs can have a significant impact in addressing DR through accessing specialist input remotely. Diabetes management requires multi-disciplinary and multi-sectoral involvement, hence good leadership is also key for advocacy and for establishing and maintaining links with stakeholders outside of eye health. 

Evidence supporting the need for health services integration in developing countries is eminent; however, literature also indicates that integration is not a blanket solution, as what works in one setting may not necessarily work in another [69]. Therefore, although the WHO has made several calls for PHC strengthening and health services’ integration, in settings where resources are limited and where PHC systems are already overburdened with various initiatives, thorough research is critical in determining how can services be integrated best without further weakening the system [52]. Moreover, inefficiencies in the broader health system will affect the delivery of eye care services at all levels of care, including PEC. Therefore, sustainable ways to either address or bypass these inefficiencies, where possible, must be considered when planning for the integration of eye care into the general health system.

## 5. Conclusions

The treatment of diabetic complications such as diabetic retinopathy is challenging and costly. This is an unnecessary burden on health systems in developing countries requiring highly skilled personnel, high technology and equipment, as well as numerous hospital visits for the patient. Conversely, the prevention of diabetes and its risk factors is simple and inexpensive. In poor settings where resources to address the diabetes burden are limited due to emphasis on infectious diseases, there is an urgent need to reorganize health systems and move away from a curative model to preventative health care. The decentralization of basic services to communities, and the development of integrated PHC-focused interventions for the prevention of diabetes and its complications is therefore essential for developing countries such as Eswatini. Investigation into how best to integrate health interventions for diabetes at the primary level should also span beyond a health worker, providing multiple treatment packages and encompassing thorough consideration of all six pillars of a health care system.

## Figures and Tables

**Table 1:** Categories of models for integrated health care in developed countries [69].

Category 1	Category 2	Category 3	Category 4
Models focused on making changes to organizations and systems	Models focused on improving patient care	Models addressing staffing needs and work ethic	Models focused on health financing and governance

## Data Availability

Not applicable.
